# Nylon clip wear and patient satisfaction in implant-retained mandibular overdenture using PEEK bar versus metal bar: a one-year randomized clinical study

**DOI:** 10.1186/s12903-026-08020-3

**Published:** 2026-03-26

**Authors:** Sara Medhat Mohamed, Doaa Amr Heshmat Rostom

**Affiliations:** 1 Lecturer of removable prosthodontics, Faculty of Oral and Dental Medicine, Modern University for Technology and Information, Cairo, Egypt; 2 Prosthodontics Department, Faculty of Dentistry, Cairo University, Cairo, Egypt

**Keywords:** Implants, Bar retained overdenture, Wear, Nylon clip

## Abstract

**Statement of problem:**

Nylon clip wear affects the retention of implant-retained overdenture.

**Purpose:**

The purpose of the present investigation was to determine the wear of nylon clips and patient satisfaction with either a cobalt-chromium (Co-Cr) metal bar or a milled polyether ether ketone (PEEK) bar in mandibular dentures supported by two implant-retained mandibular overdentures over a one-year clinical study.

**Materials and methods:**

Twenty edentulous patients were randomly divided into two groups ten patients in each group. Group I (Co-Cr metal bar with nylon clip) and Group II (PEEK bar with nylon clip). In both groups, two implants were placed in the canine–premolar region using a delayed loading protocol. The primary outcome was Nylon clip wear, it was evaluated using volumetric change and surface roughness assessment at insertion, three months, six months, nine months, and one year. Patient satisfaction was evaluated at six months and one year using a visual analog scale (VAS). Data were collected and statistically analyzed to compare the final outcomes between the two groups.

**Results:**

The metal group showed significantly higher roughness and volumetric changes with volumetric changes reached 7.49 μm ± 0.47 after 12 months (*P*=.0001) compared to the PEEK group that reached 7.21 μm ± 0.43 with no difference was found between them at 12 months (*P* = .188). Patient satisfaction scores were significantly higher in the PEEK group, regarding retention, stability, comfort, and speaking at both 6 months and 1 year (*P*=.04).

**Conclusions:**

Nylon clips showed clinically acceptable wear performance with both PEEK and metal bars after 1 year, and the PEEK bar demonstrated superior surface properties and patient satisfaction, especially in the first 6 months of the study.

**Trial registration:**

The study was approved by the Research Ethics Committee of the faculty of dentistry, Cairo University (No 94/23) and registered in the Clinical Trials.gov (NCT06909435). Trial Registration date is 27-03-2025.

**Supplementary Information:**

The online version contains supplementary material available at 10.1186/s12903-026-08020-3.

## Introduction

Over the last few years, implant-retained overdentures have demonstrated superior retention, enhanced stability, improved masticatory performance, and greater patient satisfaction while the masticatory load is distribution between the implants and the posterior residual ridge (mucosa). In compared to, Implant-Supported Overdenture: These are typically supported by four or more implants. The implants and the connecting bar bear the entire masticatory load, with little to no pressure on the soft tissues of the residual ridge [1, 2].

The choice of attachment system is one of the most crucial components of implant-retained overdenture design [3]. Retention, stability, and maintenance of the attachment systems differ, affecting the prosthesis’s functional longevity [4]. The bar attachment is a type of attachment widely used in mandibular overdentures. Bar attachments serve to splint together multiple implants, allowing for better force distribution and providing more optimal retention and stability [5, 6]. Nonetheless, bar attachments feature some complications, such as loosening of the clip, breakage or reload of a retention device, and progressive loss of retention due to mechanical wear over time. Some prostheses require more frequent maintenance, such as activation or replacement of clips to retain the preferred retention and function of the prosthesis [7, 8].

Material selection for bar attachments is important and influences the overdenture system’s performance and longevity. Bar attachments are usually made from metal, which has good strength and wear resistance [9]. Although metal bars are durable, they can make clinical adjustments difficult due to their rigidity. A high-wear-resistance material can lessen the wear of the clips used in the bar attachment, therefore increasing its service life [10, 11].

Recently, these materials have evolved with the introduction of non-metal bar materials, i.e., polyether ether ketone (PEEK). The high-performance polymer PEEK has been on the market since the late 1970s and is recognized for its favorable mechanical properties, such as chemical wear resistance, high-temperature stability, and good biocompatibility [12, 13].

PEEK is a promising material in prosthodontics for use in bar attachments, which has lower rigidity than metal with adequate strength [14]. The ability of this polymer to remain inert to biological material and resist corrosion makes it a possibility for intraoral use. In addition, compared to commonly used metal, there is limited evidence comparing the performance of PEEK bars to metal bars in terms of reducing clip wear and maintaining adequate retention over time [15]. This highlights the need for further research before PEEK can be considered a suitable bar material for attachment-retained overdentures [16, 17].

Bar clips are the key components to retain and stabilize an overdenture onto the bar attachment system. There are different clip materials, such as metal, polyoxymethylene (POM), and nylon [18–21]. Nylon (polyamide) is a strong, flexible polymer widely used in dentistry. While it offers good resistance to wear and chemicals, repeated use can reduce retention and cause discoloration. Nylon clips are the most commonly used because of the simple chairside replacement for easy maintenance [19]. The nylon clips are worn and deformed by recurring masticatory forces or the frequent insertion and removal cycles when cleaning dentures. Such wear can cause a decline in retention and stability, reducing the functional life of the overdenture [20, 21].

Existing literature indicates that the use of a PEEK bar with nylon clips in implant-retained mandibular overdentures may offer adequate surface properties during the early stages of adaptation compared to a Cobalt-Chromium (Co-Cr) metal bar, especially during the initial 6 months of use. The PEEK bar showed reduced nylon clip wear, as evidenced by lower roughness and volumetric changes. This demonstrates its potential for improved retention, especially during the initial adaptation period, as supported by recent laboratory finding [20].

The purpose of the present investigation is to determine the wear behavior of nylon clips retained by metal and PEEK bars in mandibular overdentures in order to contribute to the development of an efficient attachment system for implant-retained overdentures. This may enhance clinical success and reduce maintenance requirements for bar-retained overdentures. The null hypothesis is that no difference exists between metal and PEEK bars with respect to nylon clip wear and patient satisfaction.

## Materials and methods

The study sample size calculation is based on the research conducted by Mousa et al. [22] which utilized a minimally acceptable sample size of 10 per group, with Group I exhibiting a mean and standard deviation (SD) of 8.8 ± 0.45, while Group II showing 9.6 ± 0.55. resulting in an effect size of 1.59, with a power of 80% and a type I error probability of 0.05. The sample size calculation was performed using G*Power software version 3.1.9.7 (Heinrich-Heine-Universität Düsseldorf, Düsseldorf, Germany).

The study design was a randomized parallel group-controlled trial with an allocation ratio of 1:1. The type of randomization was block randomization. Enrollment of participants was performed by both investigators where the patients were assessed for eligibility. The first author made each participant grasped an opaque, sealed envelope from a box before bar placement step to ensure allocation concealment. Allocation sequence generation was performed by statistician. The first author completed the treatment steps and gave each patient the cleaning and maintenance instructions verbally and written. Briefly, cleansing instructions comprised brushing after meals and before sleeping. In addition, patients were instructed to wear their dentures from 14 to 16 h per day and remove them during sleeping. Patients were followed up monthly to evaluate the gingival condition by the first author. The Visual Analogue Scales evaluation was performed by the second author to ensure the blindness. Due to the nature of the intervention, blinding of the first author was not feasible. However, outcome assessment was performed by a second author who was blinded to group allocation.

Forty-six individuals were diagnosed at the outpatient clinics of the Removable Prosthodontic Department at Cairo University and MTI University, Egypt. From this group, 20 completely edentulous participants including both males and females aged 50 to 55 years with last, were selected based on specific inclusion and exclusion criteria. All participants had a minimum of 6 months post-extraction healing and similar ridge classifications to minimize bias. The final distribution was 16 males and 4 females (8 males and 2 females for each group). The study adheres to CONSORT guidelines. Each participant provided written consent before using their treatment-related radiography data in this study. The implemented radiation protection methods were in accordance with established guidelines.

All participants in this study satisfied the inclusion criteria, which include normal class I maxillomandibular verified through clinical examination and diagnostic mounting of casts on a semi-adjustable articulator., sufficient bone quantity and quality, namely in the anterior interforaminal region as evaluated by CBCT, and having an adequate inter-arch space, defined as a minimum of 12–14 mm from the ridge to the occlusal plane. Diabetic patients, patients who received radiation therapy, patients with parafunctional habits, and smokers were also excluded from the study. In accordance with the prosthetic-driven implant placement protocol, all participants received newly fabricated complete dentures prior to implant placement to standardize the baseline prosthetic conditions.

Preliminary alginate impressions (Tropicalgin, Zhermack Dental, Italy) were recorded using a perforated stock tray (Sedradent solution, Egypt) and subsequently poured into dental stone (Acrostone dental stone, Acrostone, Egypt) to create the diagnostic casts. Occlusion blocks were fabricated on the diagnostic casts following occlusal registration and try-in procedures. The artificial teeth were set in a lingualized occlusion in order to eliminate the lateral dislodging forces, improving stability, longevity and enhancing masticatory efficiency [23].

The availability of inter-arch space for the bar was evaluated on the casts using the putty-silicon-index (Panasil^®^ Putty; Kettenbach GmbH & Co. Germany) around the artificial acrylic teeth (Acrostone Cross Linked Acrylic Teeth, Acrostone, Egypt). A minimum of 13 to 14 mm of vertical space was required for a bar-supported implant retained overdenture, and finally, the upper and lower dentures that were made from heat cured acrylic resin (Acrostone Acrylic Material Heat Cure; Acrostone, Egypt) were delivered. Radiographic implant planning guides were fabricated as a duplicate from the fabricated patient mandibular denture with radiopaque markers (Gutta Percha; Meta Biomed, Korea) in the center of the teeth to guide implant positioning. The patient underwent a CBCT scan (Planmeca, Finland) while wearing the radiographic stent and was in the correct seating position.

The radiographic stent was modified into an implant surgical guide by creating perforations in the canine regions to delineate the specified surgical areas. For both groups, all patients received two 3.2 × 11.5-mm implants (Implant; Implant Dentis Co., Ltd, Korea) (in the canine-premolar region bilaterally according to bone availability in the case). All implants were placed using a surgical guide following a two-stage surgical protocol. Cover screws were screwed into the implants for three months of healing time. In the prosthetic stage, after three months of the healing period, the implants were exposed to receive healing abutments. After two weeks, the healing collars were removed, and the soft tissue was assessed for healing. Multiunit abutments (Implant; Implant Dentis Co., Ltd, Korea) were affixed at 30 N with a torque wrench. To mitigate settling effects, the multiunit abutments were retightened a minimum of 2 to 3 times at a torque of 30 N, with intervals of 10 min.

For all groups, preliminary alginate impressions (Tropicalgin, Zhermack Dental, Italy) were recorded to create a custom tray with a window at the implant site for the open tray impression technique. The long transfer copings were splinted using autopolymerizing acrylic resin (Acrostone cold cure acrylic material, Acrostone, Egypt) to ensure impression precision. A single-stage imprint procedure using putty and light body (Panasil^®^ Putty; Kettenbach GmbH & Co. Germany) was performed with silicone impression material using an open custom tray (Acrostone cold cure acrylic material; Acrostone, Egypt). The healing caps were substituted intraorally following the imprinting procedure. Analogs were screwed to the impression coping. The definitive impression was poured into cast using an extra high-strength dental stone (Elite Stone, Zhermack, Italy).

An acrylic verification jig (Duralay; Prestige Dental Products Ltd, United Kingdom) was fabricated over the impression coping using auto polymerizing acrylic resin to ensure the precision of the master cast. The intraoral single-screw test was performed to evaluate passivity.

Patients were divided into two equal groups (1:1) using computer-generated randomization. An implant-retained overdenture with a cobalt-chromium (Co-Cr) bar attachment was given to Group I (the metal bar group), and Group II (the PEEK bar group) was given an overdenture with a PEEK bar attachment.

**Fig. 1:**
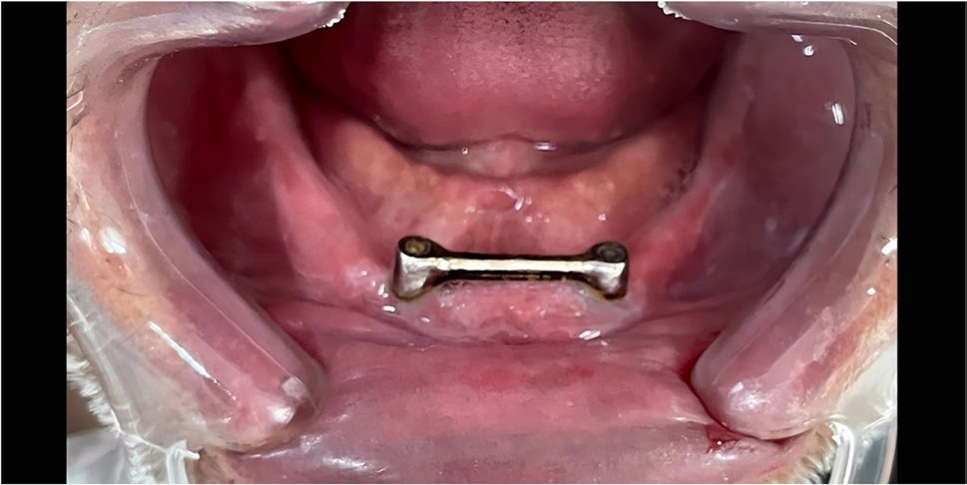
Metal bar implant retained overdenture

Regarding the Co-Cr metal bar group, in the master cast, the multiunit abutments were attached to the implant analogs. Subsequently, a wax pattern coping was tailored for each abutment and joined to a prefabricated castable bar. The bar, with a height of 2.4 mm (Rhein 83, Italy), was positioned approximately 2 to 3 mm from the anterior ridge to ensure adequate cleanliness. The wax pattern was then formed and cast in Co-Cr alloy (Magnum H50, MESA ITALIA S.R.L., Italy) (Fig. 1). Finishing was done with tungsten carbide burs, followed by rubber points and a final high-gloss polish using a cobalt-chrome polishing paste (Renfert, Germany).

**Fig. 2:**
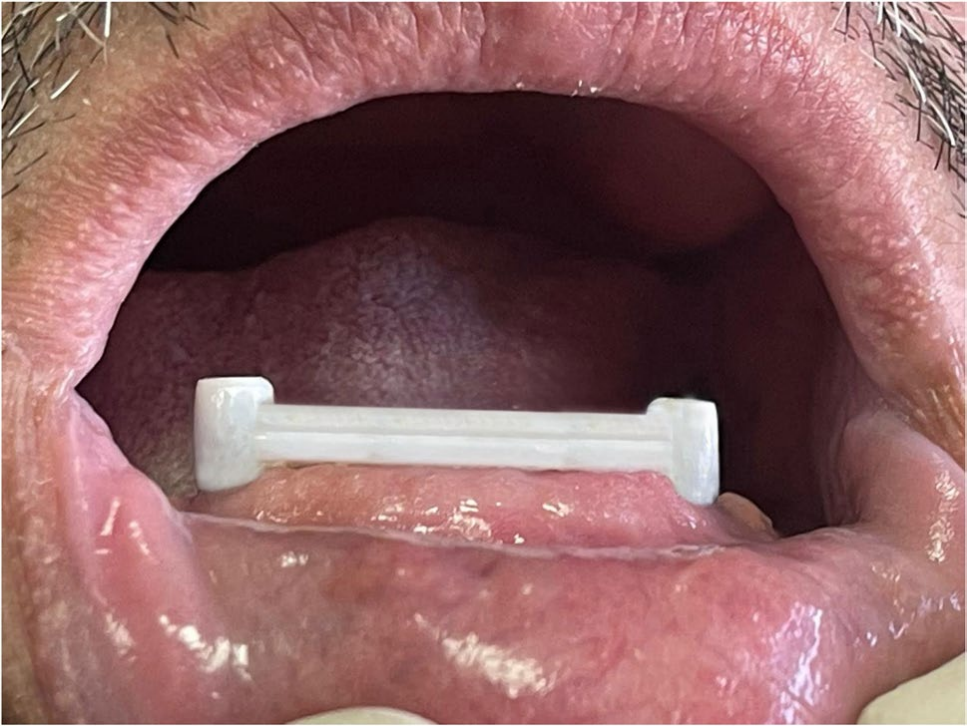
PEEK bar implant retained overdenture

Regarding the PEEK bar group, the master stone cast was scanned with an external desktop scanner to produce a Standard Tessellation Language (STL) file for a virtual model using the CAD/CAM software (Medit i500; Medit Corp, Korea). The design of the multiunit abutment and bar (Rhein 83, Italy) was selected from the CAD software library. The bar dimensions were selected according to the study bar design (Fig. 2).

After the data acquisition, the STL file was imported to the CAM part of the CAD/CAM system to mill the PEEK bar from a block of biological high-performance polymer (Bio-HPP) (Bre.CAM, Bio-HPP, Bredent GmbH & Co, United Kingdom). Polishing followed a standardized protocol using silicon polishers and a diamond-containing polishing paste (Zirkopol) to achieve a smooth surface. The screw material used was titanium alloy. The screw channel in the bar is typically precisely milled using CAD/CAM systems to match the multi-unit abutment’s screw and ensure a passive fit.

Multi-unit abutments were screwed to the implants, and the PEEK bar was screwed to the abutments. The passive fit was evaluated visually with a probe and through periapical radiography to detect misfits across all groups. The confirmation was obtained through the one-screw test, which involved securing the abutment on one side and evaluating the fit on the opposite terminal abutment.

All overdentures were seated and examined intraorally during the bar clip static pickup procedure. A relief space was created in the fitting surface of the mandibular overdenture using a large carbide bur. A clearance of approximately 2–3 mm was maintained around the bar assembly to allow for a passive seat during the direct intra-oral pick-up of the nylon clips. Condensation silicone impression material (Zitaplus, Zhermack, Italy) was utilized to occlude the area beneath the bar. The nylon clip with a diameter of 2.5 mm and a length of 3.5 mm (Rhein 83 SRL, Italy) was firmly seated over the bar, and autopolymerizing acrylic resin (Acrostone cold cure acrylic material; Acrostone, Egypt) was inserted in the relieved areas of the denture to pick up the retentive nylon clips intraorally. The acrylic resin was set, the excess material was removed, and the overdenture was delivered. To prevent tongue encroachment and ensure patient comfort, the lingual contours of the denture were carefully evaluated and polished post-polymerization to maintain their original dimensions.

To ensure the validity of the wear analysis and the longitudinal satisfaction scores, a standardized protocol for the management of retention was strictly followed. Patients were scheduled for clinical follow-up at 1, 3, 6, and 12 months. During these visits, the retentive capacity of the bar clips was assessed both subjectively (patient-reported stability) and objectively (clinical testing of resistance to vertical displacement).

Crucially, no manual activation (tightening) or replacement of the clips was performed during the 12-month study period. This non-intervention policy was essential to prevent the introduction of confounding variables that would interfere with the microscopic evaluation of material loss (wear) and to accurately reflect the natural degradation of patient satisfaction over time. Any participant who might have experienced a total loss of retention rendering the prosthesis non-functional was planned to be recorded as a ‘clinical failure’ and analyzed separately; however, all included participants completed the follow-up period with the original clips intact, allowing for a consistent one-year wear analysis.

**Fig. 3:**
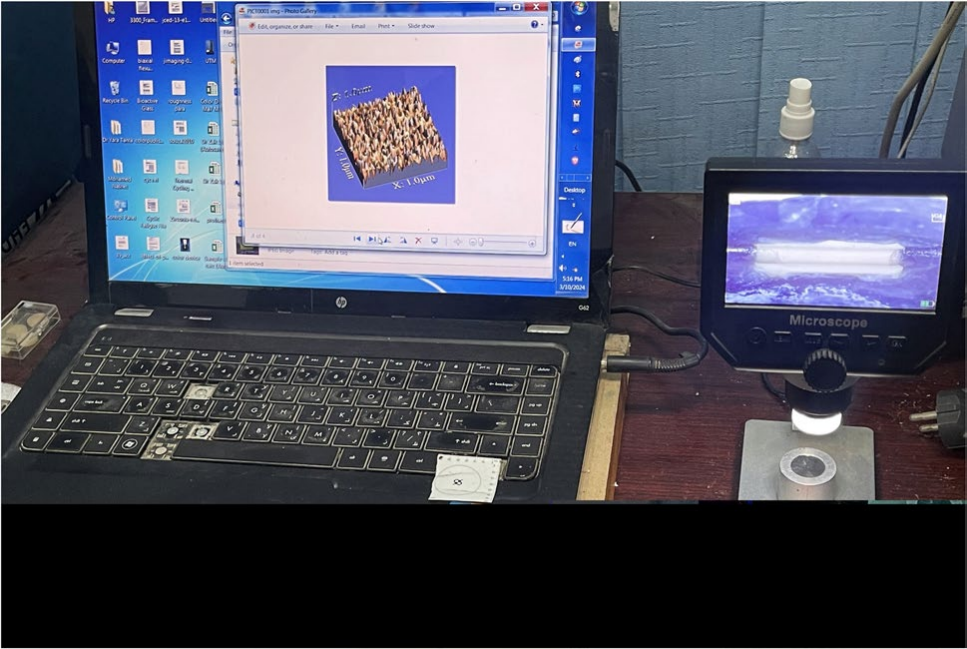
Nylon clip under microscope to evaluate roughness and volumetric changes

The wear and roughness of nylon clips were evaluated during overdenture insertion and after 3, 6, 9 months, and 1 year of clinical use using a 3D surface analyzer system [24]. The fitting surface of the denture with the nylon clip was recorded using a USB digital microscope with an integrated camera (Scope Capture Digital Microscope; Guangdong, China), which is linked to an IBM-compatible personal computer at a constant magnification of 120X, as illustrated in Fig. 3.

**Fig. 4:**
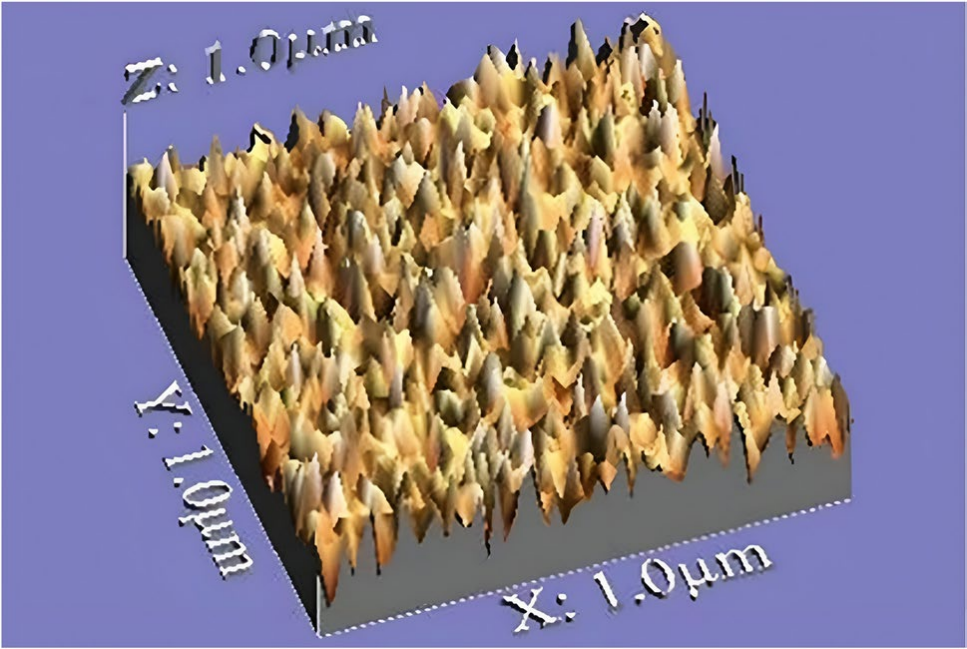
Microscopic image for nylon clip roughness and volumetric changes

The images were acquired at a resolution of 1280 × 1024 pixels. Digital microscope images were standardized for roughness assessment by cropping to dimensions of 350 × 400 pixels employing Microsoft Office Picture Manager. WSxM software version 5.0 (WSxM software; Nanotec Electronica SL, Spain) was used to measure the average heights of volumetric changes and surface roughness, which were expressed in µm [25]. A 3D image of the surface nylon clip was generated for 3D geometric analysis using a digital image analysis system (ImageJ 1.43U; National Institute of Health, USA), as presented in Fig. 4. The undamaged, unworn surface was considered as a reference point.

Patient satisfaction was evaluated using Visual Analogue Scales (VAS) by the second author after 6 months and 1 year on a scale from 1 to 10 according to the following criteria: (A) physical (stability and retention of denture), (B) functional (chewing ability, speaking ability, pain, comfort, occlusion, easiness of denture cleaning, and taste of food), and (C) aesthetic factors (appearance of dentures and self-image). This questionnaire was translated into Arabic by two certified translators and then back into English by two other certified translators. Subsequently, Face validity and clarity were assessed by ten multilingual volunteers, who evaluated both the English and Arabic versions in alternating order. No significant discrepancies in meaning or comprehension were reported.

Each patient was given a plastic card with a line scale from 1 to 10. The patient asked to mark a vertical line at the point on the horizontal line that represented his response to each question in the questionnaire.

Statistical analysis was conducted using SPSS 16^®^, GraphPad Prism, and Microsoft Excel. The data were analyzed using the Shapiro-Wilk and Kolmogorov-Smirnov tests for normality, resulting in a non-significant P-value (*P*>.05), which suggests that the data follows a normal distribution (parametric data). A comparison of two distinct groups was conducted using the independent t-test, while a comparison across two time periods was executed using the paired *t* test and was performed using Repeated Measures ANOVA, followed by Tukey’s Post Hoc test for multiple comparisons.

## Results

**Fig. 5:**
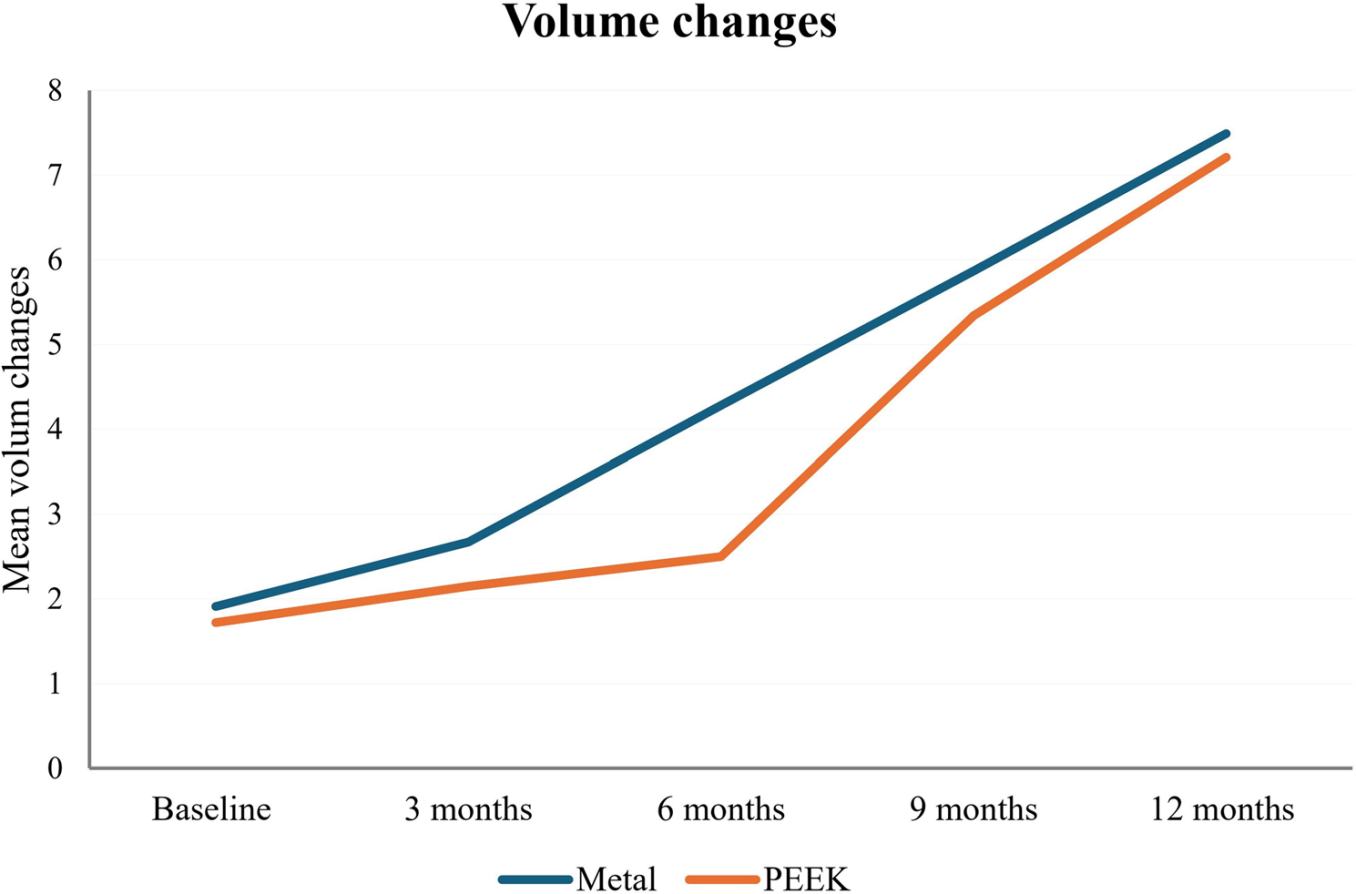
Volume changes at different time intervals in both metal and PEEK groups

Regarding the comparisons of the volumetric changes in metal and PEEK at different times. The results revealed that the metal group showed significantly higher volumetric changes than PEEK group comparing to the baseline measurements and the different assessment periods; after 3 months, after 6 months, and after 9 months (Fig. 5). On the other hand, no statistically significant difference was found between the 2 groups after 12 months (*P*=.188). Table 1While for the intragroup comparison (between different intervals), a comparison was performed using Repeated Measures ANOVA, followed by Tukey’s Post Hoc test for multiple comparisons. The analysis results revealed that there was a significant increase in volumetric changes between every 2 successive visits in metal group I, with volumetric changes increased from 1.91 μm ± 0.03 at baseline to 7.49 μm ± 0.47 after 12 months (*P*=.0001). In PEEK group II, there was a significant increase in volumetric changes between every 2 successive visits, with volumetric changes increasing from 1.72 μm ± 0.04 at baseline to 7.21 μm ± 0.43 after 12 months (*P*=.0001).

**Table 1:** Mean and standard deviation of volumetric changes in metal and PEEK at different follow-up visits

Volumetric changes	Metal		PEEK		Mean difference	Standard error difference	95% confidence interval of the difference		*P*
	Mean	Standard deviation	Mean	Standard deviation			Lower	Upper	
Baseline measurement	1.91 a	0.03	1.72 a	0.04	0.19	0.02	0.15	0.22	0.0001*
3 months	2.67 b	0.02	2.15 b	0.06	0.52	0.02	0.48	0.56	0.0001*
6 months	4.28 c	0.05	2.50 c	0.07	1.79	0.03	1.73	1.84	0.0001*
9 months	5.87 d	0.33	5.34 d	0.47	0.53	0.18	0.15	0.91	0.01*
12 months	7.49 e	0.47	7.21 e	0.43	0.28	0.20	−0.15	0.70	0.188
*P*	0.0001*		0.0001*						

Mean values having the same superscript letters in each column are statistically significantly different at *P*≤.05

Mean values having different superscript letters in each column are statistically significantly different at *P*≤.05

*Significant (*P<*.05)

**Fig. 6:**
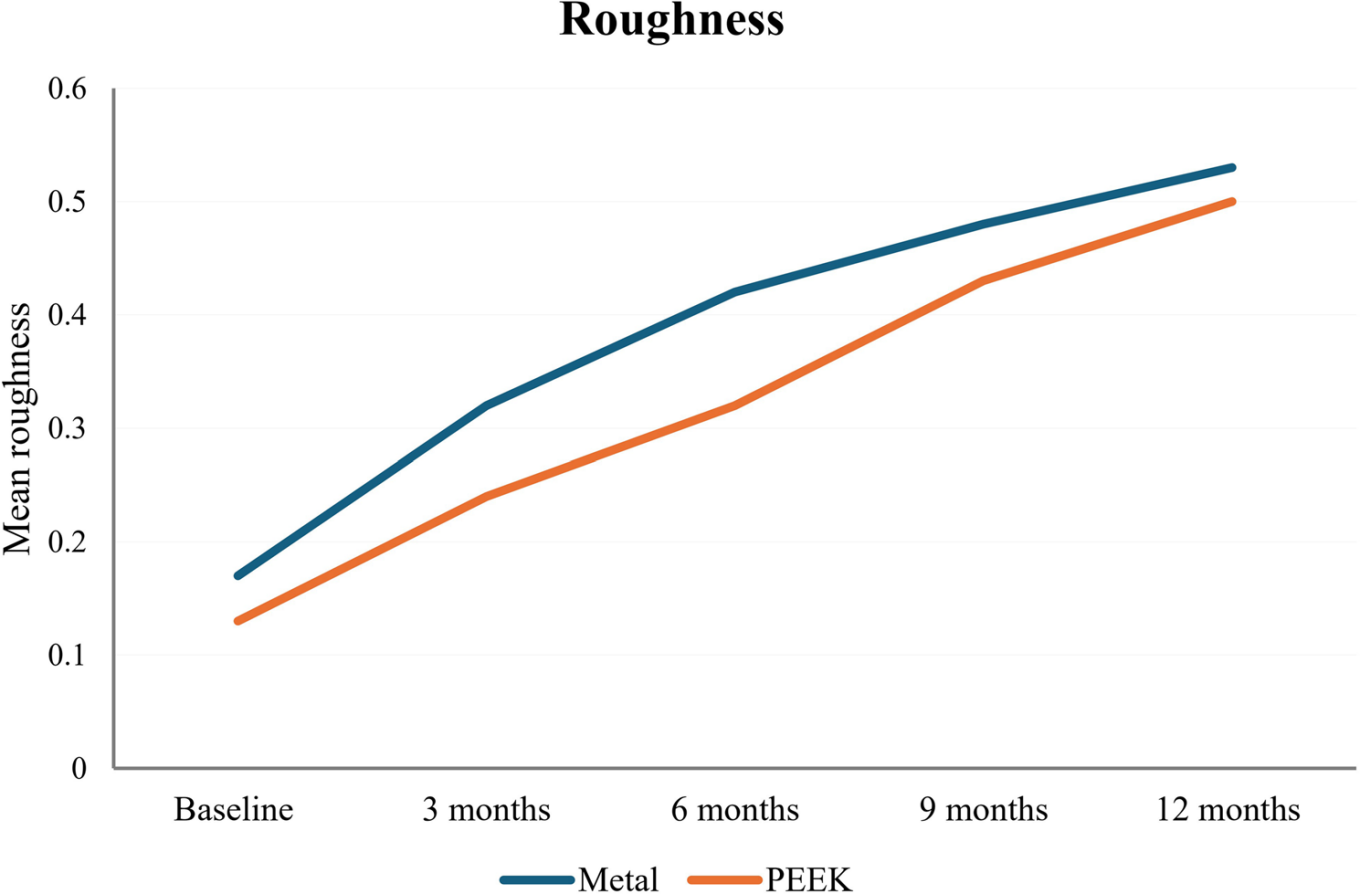
Roughness changes at different time intervals in both metal and PEEK groups

Comparisons of roughness changes in the metal and PEEK groups at different follow-up visits are shown in Table 2. An intergroup comparison between metal and PEEK was conducted using an independent t-test, which indicated that the metal group exhibited significantly greater roughness than the PEEK group at baseline, after 3, 6, and 9 months (*P*=.0001) (Fig. 6). Conversely, no statistically significant difference was found between them after 12 months (*P*=.11).

**Table 2:** Mean and standard deviation of roughness changes in metal and PEEK at different follow-up visits

Roughness	Metal		PEEK		Mean difference	Standard error difference	95% confidence interval of the difference		*P*
	Mean	Standard deviation	Mean	Standard deviation			Lower	Upper	
Baseline	0.17 a	0.00	0.13 a	0.03	−0.05	0.01	−0.07	−0.02	0.0001*
3 months	0.32 b	0.02	0.24 b	0.01	0.08	0.01	0.06	0.10	0.0001*
6 months	0.42 c	0.03	0.32 c	0.02	0.10	0.01	0.07	0.12	0.0001*
9 months	0.48 d	0.03	0.43 d	0.04	−0.05	0.01	−0.08	−0.02	0.0001*
12 months	0.53 e	0.04	0.50 e	0.04	−0.03	0.01	−0.06	0.00	0.11
*P*	0.0001*		0.0001*						

Mean values having the same superscript letters in each column are statistically significantly different at *P*≤.05

Mean values having different superscript letters in each column are statistically significantly different at *P*≤.05

*Significant (*P<*.05)

The roughness at different follow-up visits was analyzed using Repeated Measures ANOVA, with subsequent Tukey’s Post Hoc test for multiple comparisons. The results indicated that there was a significant increase in roughness between each 2 successive visits in the metal group; roughness increased from 0.17 μm ± 0.001 at baseline to 0.53 μm ± 0.04 after 12 months (*P*=.0001). In the PEEK group, there was a significant increase in roughness between each 2 successive visits; roughness increased from 0.13 μm ± 0.03 at baseline to 0.50 μm ± 0.04 after 12 months (*P*=.0001).

**Fig. 7:**
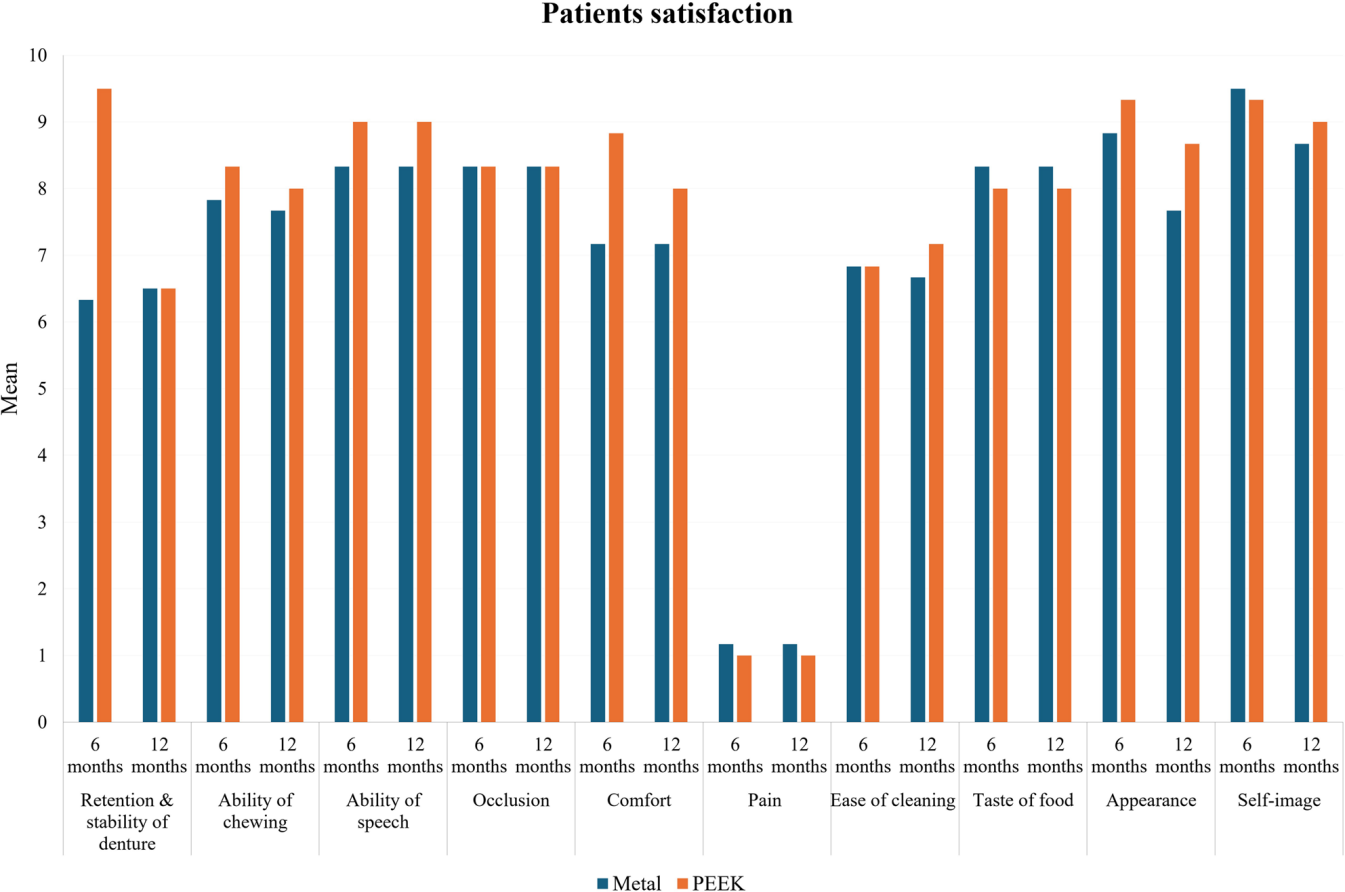
Patient satisfaction after 6 and 12 months in metal and PEEK groups

An intergroup comparison between the 2 groups regarding patient satisfaction was conducted using the Mann-Whitney test (Table 3). After 6 months, the recorded values in PEEK group were significantly higher than the recorded values of metal group regarding retention and stability of denture, the ability of speaking, and comfort (*P*=.0001), as presented in Fig. 7. On the other hand, the PEEK group revealed significantly lower results than the metal group regarding pain and taste of food (*P*=.004). While after 12 months, the PEEK group was significantly higher than the metal group regarding the ability of chewing, the ability of speaking, comfort, ease of cleaning, appearance, and self-image (*P*=.04, 0.0001, 0.01, 0.01, 0.01, 0.01, 0.01, and 0.04, respectively).

**Table 3:** Mean and standard deviation of patient satisfaction in metal and PEEK after 6 and 12 months

Roughness		Metal		PEEK		Mean difference	Standard error difference	95% confidence interval of the difference		*P*
		Mean	Standard deviation	Mean	Standard deviation			Lower	Upper	
Retention and stability of denture	6 months	6.33	0.94	9.50	0.41	−3.17	0.32	−3.85	−2.49	0.0001*
	12 months	6.50	0.71	6.50	1.08	0.00	0.41	−0.86	0.86	1.00
	*P*	0.33		0.005*						
Ability of chewing	6 months	7.83	0.62	8.33	0.47	−0.50	0.25	−1.02	0.02	0.06
	12 months	7.67	0.47	8.00	0.00	−0.33	0.15	−0.64	−0.02	0.04*
	*P*	0.06		0.06						
Ability of speaking	6 months	8.33	0.47	9.00	0.00	−0.67	0.15	−0.98	−0.36	0.0001*
	12 months	8.33	0.47	9.00	0.00	−0.67	0.15	−0.98	−0.36	0.0001*
	*P*	1.00		1.00						
Occlusion	6 months	8.33	0.47	8.33	0.47	0.00	0.21	−0.44	0.44	1.00
	12 months	8.33	0.47	8.33	0.47	0.00	0.21	−0.44	0.44	1.00
	*P*	1.00		1.00						
Comfort	6 months	7.17	0.85	8.83	0.24	−1.66	0.28	−2.25	−1.07	0.0001*
	12 months	7.17	0.85	8.00	0.41	−0.83	0.30	−1.46	−0.20	0.01*
	*P*	1.00		0.01*						
Pain	6 months	1.17	0.24	1.00	0.00	0.17	0.07	0.01	0.33	0.04*
	12 months	1.17	0.24	1.00	0.00	0.17	0.07	0.01	0.33	0.04*
	*P*	1.00		1.00						
Ease of cleaning	6 months	6.83	0.24	6.83	0.62	0.00	0.21	−0.44	0.44	1.00
	12 months	6.67	0.47	7.17	0.24	−0.50	0.17	−0.85	−0.15	0.01*
	*P*	0.06		0.06						
Taste of food	6 months	8.33	0.47	8.00	0.00	0.33	0.15	0.02	0.64	0.04*
	12 months	8.33	0.47	8.00	0.00	0.33	0.15	0.02	0.64	0.04*
	*P*	1.00		1.00						
Appearance	6 months	8.83	0.85	9.33	0.47	−0.50	0.31	−1.15	0.15	0.12
	12 months	7.67	0.47	8.67	0.94	−1.00	0.33	−1.70	−0.30	0.01*
	*P*	0.01*		0.01*						
Self-image	6 months	9.50	0.41	9.33	0.47	0.17	0.20	−0.24	0.58	0.40
	12 months	8.67	0.47	9.00	0.00	−0.33	0.15	−0.64	−0.02	0.04*
	*P*	0.01*		0.06						

Mean values having the same superscript letters in each column are statistically significantly different at *P*≤.05

Mean values having different superscript letters in each column are statistically significantly different at *P*≤.05

*Significant (*P<*.05)

Intergroup comparison between 6 and 12 months was performed using Wilcoxson signed rank. The results revealed that in the metal group, no statistically significant difference was found in patient satisfaction between 6 and 12 months in all aspects except appearance and self-image (significant decrease). In the PEEK group, no statistically significant difference was found in patient satisfaction between 6 and 12 months in all aspects except retention and stability of denture, comfort, and appearance (significant decrease).

## Discussion

In the present study, the clip efficiency and the use of different bar materials in implant-retained overdentures, which can affect the wear of prefabricated nylon caps, were evaluated. The null hypothesis was partially rejected because the results showed a significant difference in wear between metal and PEEK materials after 3, 6, and 9 months. Moreover, the results recorded a significant difference in the patient satisfaction between the two groups.

Effective pre-operative planning for the management of a complete edentulous ridge guarantees reliable outcomes and minimizes complications. It is important to prefabricate a presurgical denture as this provides a prosthetically driven implant placement concept. The patient’s inter-arch space measurement allows for the proper bar height selection and reduces the incidence of acrylic denture fracture. A verification jig was used to ensure the accurate 3D relationship of the implants and to ensure bar passivity [2, 20].

The diminished retention of the prosthesis and the frequent replacement of plastic clips are the primary challenges with bar-retained implant overdentures. Several studies have evaluated the decrease in retention forces in overdentures and suggested the underlying causes to chewing cycles, frequent insertion and removal of the prosthesis, especially by first-time denture users, and clip wear may all contribute to the loss of clip retention [19–21].

To date, almost no clinical studies have evaluated clip wear after one year of actual intraoral use; wear has only been estimated but not actually analyzed [7, 18]. However, this study measures the quantity of roughness and wear of the most commonly used nylon clip. Nylon clip is a chair-side clip that provides the quickest and easiest solution in the clinics [26].

Wear assessment was conducted by calculating the difference between the surface roughness values recorded prior to use and those obtained at each follow-up interval. Surface roughness measurements were performed using a USB digital microscope equipped with an integrated camera, and the acquired images were subsequently analyzed using specialized software. This technique offers several advantages, including low cost, ease of operation and its non-destructive nature. Nevertheless, its measurement accuracy may be influenced by operator-dependent calibration procedures and the resolution of the captured images [27]. To minimize these potential sources of variability, all measurements were performed by the same operator using identical calibration parameters at each assessment point, thereby attempting to standardize the measurement procedures.

A previous in-vitro study reported that PEEK and nylon plastic retentive clips provide equivalent retention forces after 12 months of overdenture usage. Additionally, retentive elements show a negligible decrease or a slight increase in their values after a period of 3 to 12 months [18]. The prior research indicated that the retention force values diminished to approximately 25–40% following 12 months of clinical evaluation [7, 18]. These results are in agreement with the present investigation since nylon clips in both groups reveal an increase in roughness and wear over time, but the metal group showed significantly higher volumetric changes than the PEEK groups.

The findings of this study indicated no statistically significant difference in volumetric changes between the groups after 12 months. After an extended period of clinical use, the retention force values stabilized, showing no variation attributable to the roughness of plastic components, which leads to more friction [28]. Furthermore, the oral environment, saliva composition, and temperature may also exert influence [25].

The roughness surface cut off 0.2 μm is the threshold for plaque accumulation and material wear in dentistry. We have now incorporated this benchmark. While both groups slightly exceeded this threshold at 3 months, the PEEK group (0.24 μm) was much closer to the ideal value than the Metal group (0.32 μm). This suggests that PEEK’s superior polished surface and wear resistance may maintain a smoother interface over time, which explains the reduced volumetric wear of the clips in that group.

Regarding PEEK Group, with an elastic modulus similar to human bone, the PEEK bar provides a ‘stress-breaking’ effect. Its inherent resilience allows it to absorb a portion of the rotational energy, thereby reducing the peak deformation forces transmitted to the nylon clip. While in the metal (Co-Cr) Group, the extreme rigidity of the Cobalt-Chromium bar offers no stress relief, concentrating the full masticatory load onto the nylon clip interface. This leads to higher localized heat and friction, accelerating the volumetric loss and surface roughening identified in our results [20]. 

An in-vitro study evaluated the retention of various clip materials in relation to different bar materials [21]. The findings indicated that the highest mean retention value was associated with the PEEK bar and PEEK clip, followed by the metal bar and PEEK clip, and the PEEK bar and POM clip. The lowest mean retention value was noted with the metal bar and POM clip [21]. Abdraboh et al., [17] conducted a one-year clinical trial demonstrating that PEEK bars showed reduced wear on plastic clips due to their lower rigidity compared to Co-Cr. This is in agreement with the present study’s statistical analysis, which demonstrates increased roughness and volumetric changes in the nylon clip with the metal bar compared to the PEEK bar after one clinical year.

An in-vitro study in 2024 reported the relation between the change in the retentive value of the bar clip to their different moduli of elasticity; the elastic modulus value of the zirconia is 1400–2500 MPa, the elastic modulus of BioHPP is about 4000 MPa while the plastic clips resin has an acceptable rigidity, toughness, and a 305 MPa modulus of elasticity. PEEK has shock-absorbing action offered during occlusion and chewing force, and the PEEK esthetic advantage is that no metallic shadow is shown underneath the denture, in addition to its good wear resistance [20].

Another advantage of the PEEK material is its low tendency to colonize bacteria over its surface. According to Wiessner et al. [29], microbial buildup is more pronounced on polymer-based bars (PEEK and BioHPP) than on metal and zirconia bars. Conversely, some studies indicate that microbial adherence on PEEK bars is comparable to or less than that found on zirconia and metal bars. These findings align with those of Barkamo et al., [30] who found no significant differences in microbial accumulation between PEEK and titanium bars; however, they noted that increased roughness of PEEK bars significantly enhanced microbial adherence. Further research is required to assess whether polymer-based bars enhance surface roughness over time and exhibit increased susceptibility to microbial adhesion [16, 29, 30].

Previous literature recommends that most of the investigated clip materials showed satisfactory retention forces with time. Other factors, rather than retention, may govern clinical treatment options, as the ease of usage for the prosthodontist may be an important factor when selecting the clip material. A nylon clip could still be used instead of the PEEK, but the PEEK clip needs scanning and designing, while the nylon clip is much cheaper than any alternative and readymade [31].

The current study evaluated patient satisfaction after 6 months and one year, and the result showed that the PEEK group was significantly higher than the metal group regarding the ability of chewing and speaking, comfort, ease of cleaning, appearance, and self-image. Regarding retention and stability of the denture, the PEEK group exhibited a significantly higher performance compared to the metal group, and this explains the high mean value of the roughness and volumetric change in the metal group more than in the PEEK group.

Previous literature evaluated both groups according to physical, functional, and aesthetic factors using a visual analog scale (VAS). The results showed that the patients in the (BioHPP) bar group were more satisfied than patients in the (Co-Cr) bar group. The comfort was more significant in the (BioHPP) group, which was attributed to the minimal bar weight of BioHPP [22, 32]. Contrary to our result, in the comparison between 6 and 12 months regarding PEEK group satisfaction, comfort significantly decreased after one year, and this may be due to increases in clip wear roughness at the end of the study [32]. Moreover, the both group after 6 months showed higher appearance scores due to highly polished surfaces of the overdentures, then decline at 12 months was likely due to surface staining or slight discoloration of the overdenture material over time.

The statistical results in the current study during the first 6 months revealed a remarkable volumetric change in the mean value of the PEEK, which is lower than in the metal group, and after 6 months, patient satisfaction analysis showed that the PEEK group was significantly higher than metal group regarding retention and stability of the denture, to speak, and comfort. This might be due to the good stress distribution of the PEEK and nylon clip together. The material hardness of the bar and the associated clip, together with the patient’s muscle strength during centric and eccentric mandibular movements, should be considered in case selection to ensure the longevity of rehabilitation [26, 33].

This study was limited by its one-year follow-up duration, evaluation of a single bar design, and inclusion of both males and females, with the higher biting force of the males affecting the clip wear. In addition, the study evaluated only single bar and clip system brand that may not reflect performance variations with other commercial systems.

### Limitations

The current study was limited to evaluating mechanical wear and patient satisfaction. However, the appropriate selection of a bar-clip system is a multifactorial decision influenced by several biological parameters—such as peri-implant soft tissue health and plaque accumulation—as well as factors related to cost, time, and maintenance frequency. In addition, different fabrication techniques were used for the two bar materials (milled PEEK versus cast Co–Cr), which may have influenced surface properties, fit, and consequently nylon clip wear and retention. These aspects require further research to be thoroughly evaluated.

## Conclusions

Under limitations of the study, the use of a PEEK bar with nylon clips in implant-retained mandibular overdentures provide advantage reliable surface property and patient satisfaction compared to a Cobalt-Chromium (Co-Cr) metal bar with nylon clip, especially during the initial 6 months of use.

### Recommendation

Future studies should focus on the long-term evaluation of bar-clip systems, with particular emphasis on the combined effects of material properties, oral environment, and patient-specific factors. In addition, future research should compare digital impression techniques and digital workflows used in the construction of bar attachments with conventional fabrication methods.

### Clinical implications

The findings of this study have several significant implications for clinical prosthodontics. First, the use of CAD/CAM milled PEEK bars offers a more ‘user-friendly’ biological interface for nylon clips compared to cast metal bars, primarily due to PEEK’s lower surface roughness and its ability to absorb functional stresses. For the clinician, this may translate into reduced chair-side time spent on frequent clip activations or replacements during the first 6 to 12 months of function. Second, from a patient satisfaction score – particularly regarding comfort and aesthetics – suggest that PEEK is a superior alternative for patients who are sensitive to the weight or metallic taste of traditional alloys. Finally, the integration of PEEK into a fully digital workflow allows for high-precision fabrication, making it a viable and efficient material for long-term implant-retained overdenture therapy, especially in cases where a stress-breaking effect is desired to protect the underlying implants and bone.

## Supplementary Information

Supplementary Material 1.

## Data Availability

The datasets used and/or analyzed during the current study are available from the corresponding author on reasonable request.

## Abbreviations

Co-Cr: Cobalt-Chromium
PEEK: Polyether ether ketone
POM: Polyoxymethylene
VAS: Visual Analogue Scale
