# Supplementary material for: Nylon clip wear and patient satisfaction in implant-retained mandibular overdenture using PEEK bar versus metal bar: a one-year randomized clinical study
Source: BMC Oral Health. 2026 Mar 26;26:628. doi: 10.1186/s12903-026-08020-3 (PMC13063860; doi:10.1186/s12903-026-08020-3)
Supplement: Supplementary file 1 — Supplementary Material 1. [file 12903_2026_8020_MOESM1_ESM.docx]

**CONSORT Flow Diagram**

Lost to follow-up (patients did not answer the calls) (n=2)

Discontinued intervention (n=0)

Lost to follow-up (patients did not answer the calls) (n= 2)

Discontinued comparator (n=0)

Allocated to PEEK bar (n=10)

♦ Received allocated intervention (n=10)

♦ Did not receive allocated intervention (n=0)

Analysed (n=8)
♦ Excluded from analysis (n=0)

Analysed (n=8)
♦ Excluded from analysis (n=0)

Analysis

Follow-Up

Allocated to metal bar (n=10)

♦ Received allocated Comparator (n=10)

♦ Did not receive allocated comparator (n=0)

Assessed for eligibility (n=46 patient)

**Randomized (n= 20)**

Allocation

Excluded (n= 26)

♦  Not meeting inclusion criteria (n= 18)

♦  Declined to participate (n=4)

♦  Other reasons (n=4)

Enrollment
